# PODNL1 promotes cell proliferation and migration in glioma via regulating Akt/mTOR pathway

**DOI:** 10.7150/jca.46901

**Published:** 2020-08-27

**Authors:** Yibo Geng, Pengcheng Zuo, Xiao-ou Li, Liwei Zhang

**Affiliations:** 1 Department of Neurosurgery, Beijing Tiantan Hospital, Capital Medical University, Beijing, China.; 2 China National Clinical Research Center for Neurological Disease, Nan Si Huan Xi Lu 119, Fengtai District, Beijing 100070, China.

**Keywords:** podocan-like protein 1, glioma, epithelial-mesenchymal transition, Akt/mTOR pathway

## Abstract

**Background and Aims:** Emerging studies have determined that the small leucine-rich proteoglycan (SLRP) family can aggravate tumor progression. However, the biological function of podocan-like protein 1 (PODNL1), a novel member of the SLRP family, has not been investigated. Therefore, our study focused on the function and regulatory mechanism of PODNL1 in glioma.

**Methods:** Both the Gene Expression Profiling Interactive Analysis (GEPIA) and the Chinese Glioma Genome Atlas (CGGA) database were used to analyze the expression level and survival risk of PODNL1 in glioma. Quantitative real-time polymerase chain reaction (qRT-PCR) and Western blot were applied to detect the mRNA and protein expression, respectively. Celltiter-Glo and colony formation assays were used to evaluate cell proliferation. Migration capacity was measured by Transwell and wound healing assays. Flow cytometry was utilized to assess the apoptotic rate.

**Results:** The expression of PODNL1 predicted the poor prognosis in glioma patients. Silencing of PODNL1 inhibited cell proliferation, migration, and induced epithelial-like phenotype. In addition, knockdown of PODNL1 also induced cell apoptosis. Moreover, the cell growth and migration inhibited by PODNL1 knockdown could be partially rescued with Akt activator. Conversely, PODNL1 overexpression promoted cell growth and migration, which were suppressed by Akt inhibitor.

**Conclusions:** PODNL1, a promising predictive indicator of poor prognosis, resulted in greater proliferation, migration and epithelial-mesenchymal transition (EMT) process. Moreover, PODNL1 promoted aggressive glioma behavior by activating Akt/mTOR pathway, providing a novel therapeutic target for glioma.

## Introduction

Glioma is one of the most common intracranial malignant tumors in adults, characterized by aggressive phenotype and high mortality 1. Although there are sustained efforts to treat glioma, including maximal safety resection, chemo-radiotherapy and some pathway inhibitors, the prognosis remains dismal 2. To improve prognosis, many studies focus on the aberrantly expressed genes and underlying mechanism of glioma, which provides precise targets for glioma treatment.

PODNL1, as a member of SLRP, has been found to highly express in high-grade glioma 3 and be a survival risk to glioma patients 4 according to previous studies. Although these results suggest that PODNL1 is a biomarker for glioma, the biological functions and the underlying mechanism of PODNL1 in glioma remain unknown.

Our study aimed to identify the function of PODNL1 in glioma progression. Firstly, we analyzed the expression levels of PODNL1 in WHO II-IV grade glioma tissues and compared the overall survival time between high and low PODNL1 expression groups through The Cancer Genome Atlas (TCGA) and CGGA databases. Next, our study suggested that for the first time and as far as we knew, suppression of PODNL1 remarkably inhibited glioma cell proliferation, migration, EMT, and induced apoptosis. Conversely, most of these phenotypes showed the opposite trend in PODNL1 overexpression cells. Moreover, the effects of cell growth and migration caused by PODNL1 knockdown and overexpression could be partially rescued by Akt activator and inhibitor, respectively. These results indicated that PODNL1 aggravated glioma malignant behavior by regulating Akt/mTOR pathway.

## Materials and Methods

### Public databases analysis

Differential expression and prognosis of PODNL1 in glioma tissues were evaluated by analyzing the Gene Expression Profiling Interactive Analysis (GEPIA) dataset 5. When analyzing the CGGA database, we downloaded the original data (Table S1) from the website and then excluded the patients without overall survival time, isocitrate dehydrogenase (IDH) status, or histology. Finally, 314 patients were included in our further analysis.

### Reagents and antibodies

SC79 and MK-2206 (Selleck, Pittsburgh, PA, USA) were dissolved in dimethyl sulfoxide (DMSO, Sigma) for *in vitro* experiments. Primary antibodies used in Western blot and immunohistochemistry were GAPDH (abm, Zhenjiang, Jiangsu, China), PODNL1 (Thermo Fisher Scientific, Inc., St. Louis, MO, USA), E-cadherin (Cell Signaling Technology, Danvers, MA, USA), N-cadherin (BD Biosciences, San Jose, CA, USA), Vimentin (Thermo Fisher Scientific), phospho-mTOR (Ser2448, Cell Signaling Technology), mTOR (Cell Signaling Technology), phospho-Akt (Ser473, Cell Signaling Technology), Akt (Cell Signaling Technology), Bax (Cell Signaling Technology), Bcl-2 (Cell Signaling Technology) and cleaved Caspase-3 (Cell Signaling Technology).

### Cell culture

The human malignant glioma cells (U87, U251, and U343) were kindly gifts from Dr. Xin Chen in Capital Medical University, and HEB astrocytes were purchased from Beijing winter song Boye Biotechnology (Beijing, China). Above-mentioned cell lines were cultured in Dulbecco's modified Eagle's medium (DMEM, Corning, NY, USA) with 10% fetal bovine serum (FBS) (HyClone, Logan, UT, USA) and 1% penicillin and streptomycin (Gibco, Rockville, MD, USA) in a humidified atmosphere containing 5% carbon dioxide. Patient-derived diffuse intrinsic pontine glioma cell lines TT150630 and TT150714 were cultured as previously described 6. All cell lines used in our study were confirmed to be negative for mycoplasma.

### Construction of plasmids

For the preparation of lentiviral shRNA plasmids targeting PODNL1, three different targeting sequences (Lv-shP1: GCCCTTACAAAGCTACCAGCC, Lv-shP2: TTCGAGTGTTCCCGGACAACA, Lv-shP3: GCAAGAGATCCACATTTCTGC) were synthesized and inserted into the FUGW-H1 lentiviral plasmid as previously described 7. To package lentivirus, the lentiviral constructs were co-transfected using Neofect (Neofect biotech, Beijing, China) with plasmids of psPAX2 and pMD2.G into 293FT cells. The following steps were mentioned before 8. PiggyBac-CAG-EGFP and Pbase (to provide transposase) plasmids were gifts from Dr. Jie Na and Xiaohua Shen at Tsinghua University. PiggyBac-CAG-IRES-EGFP (PB-C) and piggyBac-CAG-PODNL1-IRES-EGFP (PB-OE) were constructed by inserting IRES or PODNL1-IRES into the *BamH1* restriction site on the backbone using In-Fusion Cloning Kit (abm).

### RNA isolation and qRT-PCR

TRIzol (Invitrogen, Carlsbad, CA, USA) was used to isolate the total RNA from cells. According to the supplier's instructions, the total RNA was reversely transcribed into cDNA using the commercial kit (abm). Bio-rad CFX384 (Bio-Rad, Hercules, CA, USA) and SYBR Green (CWBIO, Beijing, China) were adopted for qRT-PCR. Relative expression of PODNL1 was evaluated using the 2^-ΔΔCt^ method and normalized to GAPDH. The specific primer sequences information is shown below (Table 1).

### Cell viability

2,000 cells/well were plated in 96-well plates in triplicates. At 0, 12, 24, 36, 48 and 72 h, cell viability was evaluated by Celltiter-Glo assay (Promega, Madison, WI, USA) and the signal was achieved by a TECAN Infinite 2000 plate reader (TECAN, Maennedorf, Zürich, Switzerland).

### Colony formation assay

400 cells/well were cultured in triplicates in 6-well plates and incubated at 37 °C. After 14 days, cells were fixed by methanol for 30 minutes and stained with 0.5% crystal violet for 30 minutes. Subsequently, the plates were washed in water to remove excessive crystal violet. The plates were scanned using an electronic scanner (Fuji Xerox, Tokyo, Japan).

### Apoptosis assay

Flow cytometry was performed to evaluate cell apoptosis. Cells were gently harvested and stained with Annexin V-PE Apoptosis Detection Kit with 7-AAD (Beyotime Biotechnology, Shanghai, China) according to the manufacturer's instruction. Finally, flow cytometric analysis was performed by CytoFLEX (Beckman Coulter, Inc, Kraemer Boulevard Brea, CA, USA) in compliance with the manufacturers' instructions.

### Transwell assay

5×10^4^ transfected cells suspended in serum-free DMEM were seeded in the upper chambers of the Transwell system (Corning, NY, USA). Then, the DMEM containing 10% FBS was added to the lower chamber to induce cell migration. After 24 h, the upper chambers were fixed by 4% paraformaldehyde for 30 minutes and then stained with 0.5% crystal violet for 10 minutes. Following washing with running water gently, the cells on the upper surface of the membrane were removed by a cotton swab. The membranes were captured under a microscope (Zeiss, Jena, Germany).

### Wound healing assay

4×10^5^ cells/well were seeded into 6-well plates and incubated 24 hrs. Wounds were created by scratching the cell layer with a 1000 ul pipette tip. Then, cells were cultured in the DMEM containing 2% FBS for another 24 h and images were captured under a microscope (Zeiss).

### Western blot

RIPA lysis buffer (25 mM pH 7.6 Tris-HCl, 150 mM sodium chloride, 1% NP-40, 1% sodium deoxycholate, 0.1% sodium dodecyl sulfate) was utilized to extract total protein from different groups of cells. Following quantification using Pierce BCA Protein Assay Kit (Thermo Fisher Scientific), equal amounts of protein were loaded and separated by SDS-PAGE electrophoresis and transferred to PVDF membranes (Thermo Fisher Scientific). Then, the membranes were blocked with 5% bovine serum albumin (BSA) for 1 hr and then incubated with primary antibody (1:1000) at 4 °C overnight. Membranes were then incubated with horseradish peroxidase (HRP)-conjugated secondary antibody (CWBIO) at room temperature for 1 hr. Protein bands were visualized with the enhanced chemiluminescence detection kit (Thermo Fisher Scientific) and the ChemiDoc Touch imaging system (Bio-rad).

### Animal experiments

All *in vivo* experiments were approved by the Committee on the Ethics of Animal Experiments of Beijing Tiantan Hospital. 10^6^ active U87 cells stably expressing H1 or shPODNL1-3 were subcutaneously injected into the four-week-old BALB/C nude mice (Beijing Vital River Laboratory Animal Technology Co., Ltd., Beijing, China). After 30 days, the mice were euthanized and the xenografts were excised, weighed and fixed by formalin.

### Hematoxylin-eosin staining (H&E) and immunohistochemistry (IHC)

Formalin-fixed paraffin-embedded sections of xenografts were deparaffinized with xylene and graded ethanol. For H&E, the sections were stained with hematoxylin and eosin (Sangon Biotech) according to the standard protocol 9. For IHC, the sections were incubated with 0.3% H_2_O_2_ in ethanol for 30 min, followed by incubation with anti-PODNL1 overnight at 4 °C. Subsequently, the sections were visualized using 3,3'-diaminobenzidine (DAB, Beyotime Biotechnology) and co-stained with hematoxylin (Sangon Biotech). Finally, the sections were mounted with neutral gum (CWBIO) and captured by the microscope (Zeiss).

### Statistics

Statistical analysis was performed using GraphPad Prism 7.0 (GraphPad Software, Inc., La Jolla, CA, USA). ImageJ software was used for the quantification of immunoblot bands. Data from three independent replicate experiments in our study were expressed as mean ± standard deviation (SD). The Student's *t*-test or one-way analysis of variance (ANOVA) was utilized to compare differences between two and three (or more) groups, respectively. Kaplan-Meier method and log-rank test were carried out for survival analysis. *P* < 0.05 was considered to indicate statistical significance.

## Results

### Upregulation of PODNL1 was correlated with poor prognosis in glioma

**Figure 1:**
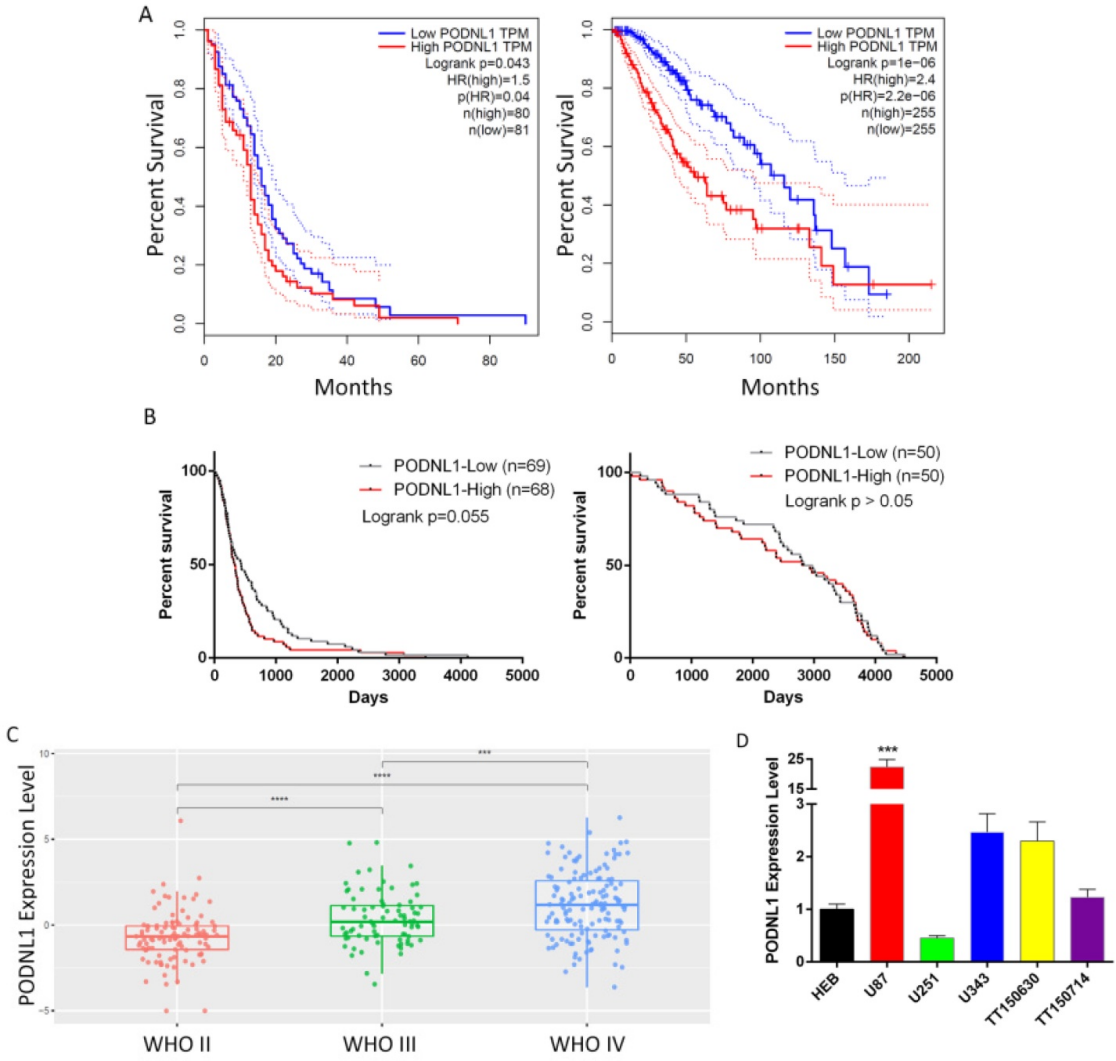
** Clinical significance of PODNL1 expression in glioma patients. A,** The Kaplan-Meier survival curve suggested that high PODNL1 expression predicted a shorter overall survival for low-grade glioma (right) and glioblastoma (left) patients in the TCGA database. **B,** The survival curves of low-grade glioma (right) and glioblastoma (left) patients in the CGGA database. **C,** The PODNL1 expression was positively correlated with WHO grade II-IV. **D,** The PODNL1 is upregulated in most glioma cell lines compared with normal astrocytes. (***P < 0.001, ****P < 0.0001).

To evaluate the clinical significance of PODNL1 in glioma patients, we firstly analyzed the overall survival time through the two independent databases, TCGA and CGGA. Both showed that the expression of PODNL1 was negatively correlated with overall survival (Figure 1A, B). Strikingly, in the CGGA database, the median survival time of the high PODNL1 expression group was 4.3-fold compared with the low PODNL1 expression group (Figure 1B, Table S1). Moreover, PODNL1 expression was significantly along with the WHO grade (Figure 1C). In addition, we noticed that PODNL1 was highly expressed in IDH wildtype (IDH-Wt) status (Figure S1A) which was well accepted as a risk factor of glioma 10, 11. To exclude the influence of IDH mutation status, we performed the survival curve in the IDH mutant (IDH-Mu) or IDH-Wt group, respectively. As expected, elevated PODNL1 expression predicted poor overall survival of glioma in both the IDH-Mu and IDH-Wt groups (Figure S1B, S1C). Furthermore, we detected the expression level of PODNL1 in an astrocyte cell line and several human glioma cell lines, which suggested that most of the glioma cells highly expressed PODNL1 whereas one fifth expressed lower than astrocytes (Figure 1D).

### PODNL1 promoted proliferation and motility capacities of glioma cells

**Figure 2:**
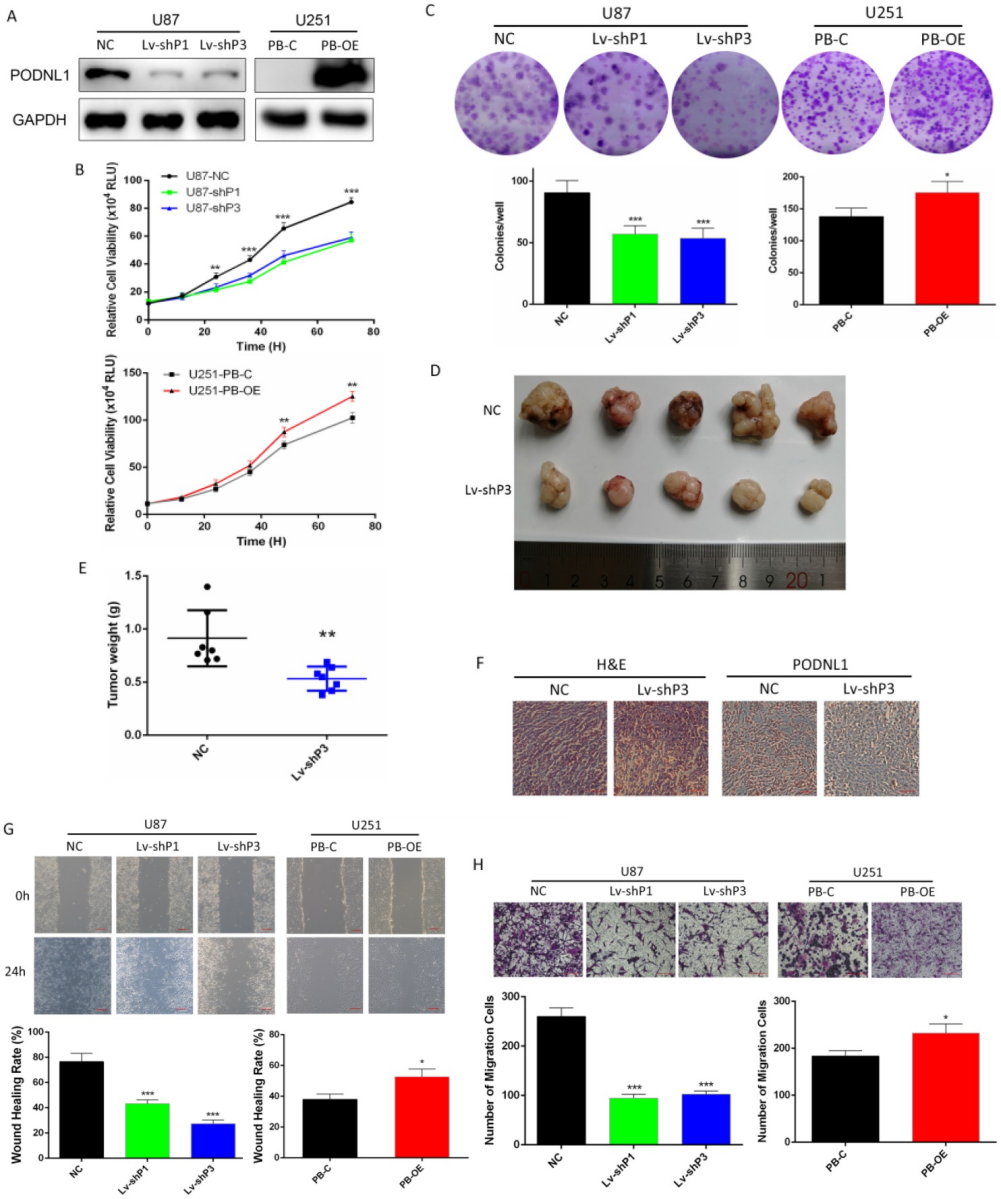
** PODNL1 promoted glioma cell proliferation and migration. A,** Knockdown and overexpression efficiencies were detected by Western blot in U87 and U251, respectively.** B, C,** Effects of PODNL1 knockdown and overexpression on glioma proliferation were determined by Celltiter-Glo** (B)** and colony formation assays** (C)**.** D, E,** The representative image** (D)** and weight** (E)** of xenograft tumors derived from subcutaneous implantation of U87 cells infected with NC or Lv-shP3.** F,** Representative immunohistochemical images of H&E and PODNL1. Scale bar, 200 µm.** G, H,** Effects of PODNL1 knockdown and overexpression on glioma migration were determined by wound healing (**G,** scale bar, 200 µm) and Transwell assays (**H**, scale bar, 500 µm). (*P < 0.05, **P < 0.01, ***P < 0.001).

Due to U87 and U251 cell lines represented the high and low endogenous expression of PODNL1, they were selected for our further investigations. To deepen study the biological function of PODNL1 in glioma, three different shRNAs specifically targeting PODNL1 were employed to achieve knockdown in U87 cells. Moreover, we also performed PODNL1 overexpression in U251 cells using the piggyBac system. The efficiencies of PODNL1 knockdown and overexpression were assessed by qRT-PCR (Figure S2A, S2B). Then, the two most efficient shRNAs (Lv-shP1, Lv-shP3), as well as PB-OE were selected and verified by Western blot (Figure 2A). Subsequently, Celltiter-Glo and clonogenic assays demonstrated that suppression of PODNL1 significantly decreased the proliferation and colonies number (Figure 2B, C). Moreover, xenograft mouse models were established by injecting U87 cells stably transfected with the H1 or Lv-shP3 plasmid into nude mice. Suppression of PODNL1 dramatically reduced the tumor weight compared with the empty vector control (Figure 2D, E). Immunohistochemistry verified PODNL1 knockdown in the xenograft tumors (Figure 2F). Furthermore, cell migration was remarkably suppressed in PODNL1-knockdown groups, compared with the NC group (Figure 2G, H). Conversely, PODNL1 overexpression greatly promoted the proliferation and clonogenic formation (Figure 2B, C), as well as increasing migration ability (Figure 2G, H).

### Knockdown of PODNL1 increased glioma cells apoptosis

**Figure 3:**
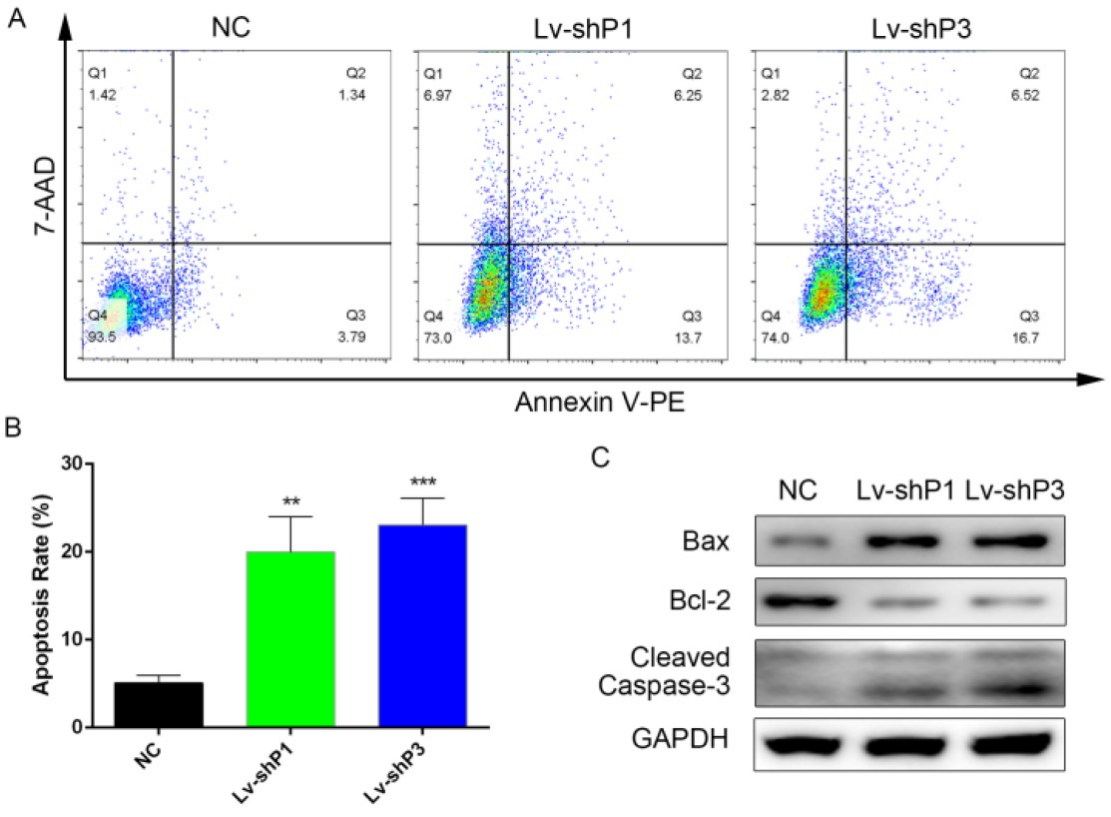
** Knockdown of PODNL1 induced glioma cell apoptosis. A,** Apoptotic cells were detected by flow cytometry. **B,** Statistical graph showed the apoptosis rate induced by PODNL1 knockdown. The number of Q2 + Q3 was defined as apoptotic cells. **C,** Western blot was used to detect the level of proteins related to apoptosis (Bcl‐2, Bax, Cleaved Caspase-3). (**P < 0.01, ***P < 0.001).

To further explore the function of PODNL1 in glioma progression, flow cytometry was performed to assess the cell apoptosis rate among the PODNL1 knockdown and overexpression groups. The results indicated that knockdown of PODNL1 significantly increased the percentage of apoptotic cells compared with the NC group (Figure 3A, B). Consistently, Bcl-2 was decreased while Bax and cleaved Caspase-3 were increased following PODNL1 knockdown in U87 cells. Whereas, PODNL1 overexpression had little effect on apoptosis (Figure S3).

### PODNL1 triggered EMT in glioma cells

**Figure 4:**
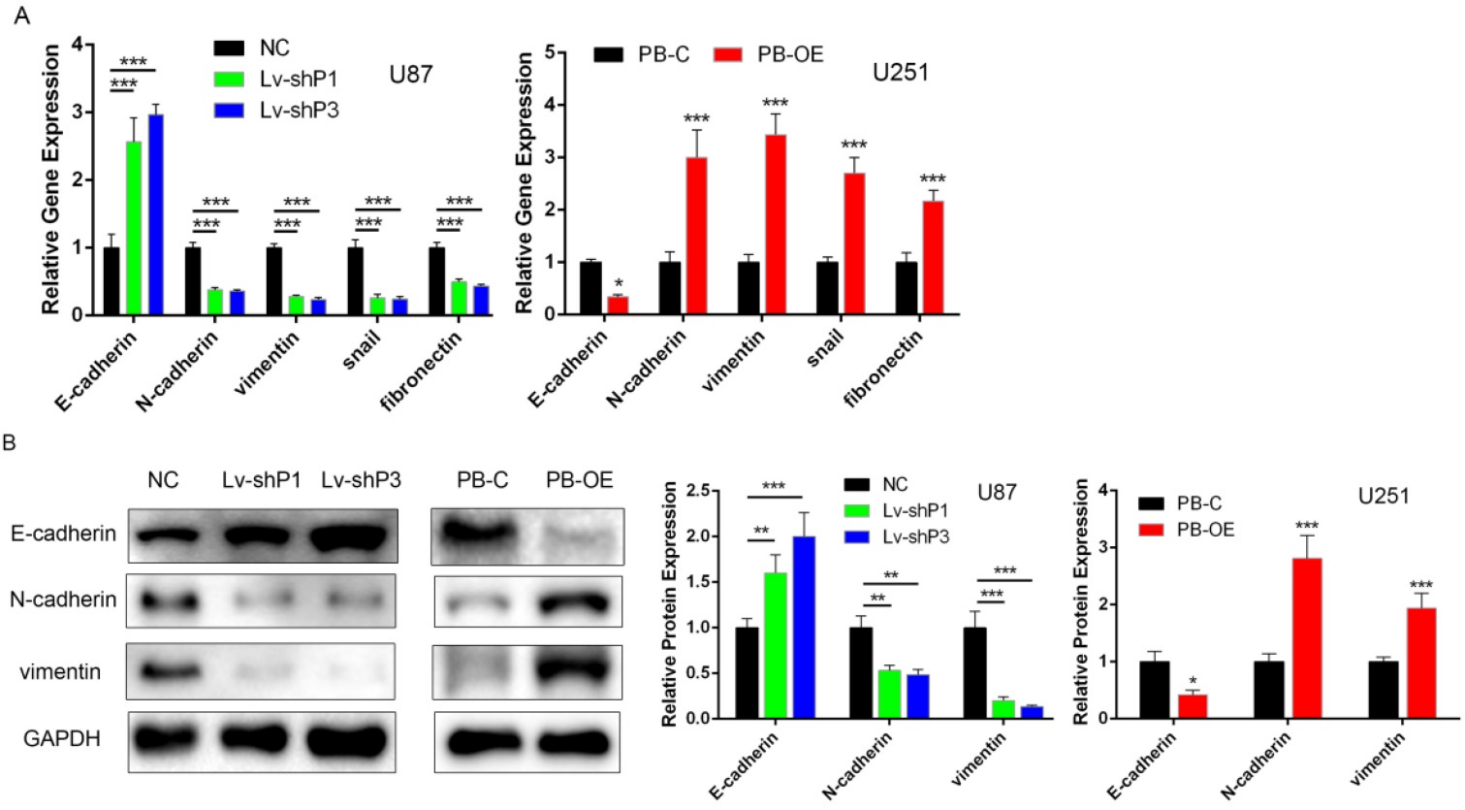
** PODNL1 triggered EMT in glioma cells. A,** mRNA expression levels of EMT markers after PODNL1 knockdown (left) or overexpression (right) evaluated by qRT-PCR. **B,** Protein expression levels of EMT markers after PODNL1 knockdown or overexpression detected by Western blot. (*P < 0.05, **P < 0.01, ***P < 0.001).

Recently, several studies have suggested that the SLRP family contribute to EMT. Considering PODNL1 promoted glioma cell migration (Figure 2D, E), we wondered if PODNL1 could regulate EMT. Hence, qRT-PCR and Western blot were performed to detect the expression of EMT markers. The results revealed that PODNL1 knockdown remarkably reduced the expression of vimentin, N-cadherin, snail and fibronectin, while increased the expression of E-cadherin (Figure 4A). Conversely, PODNL1 overexpression resulted in a mesenchymal-like molecular phenotype (Figure 4A). Furthermore, the expression levels of E-cadherin, N-cadherin and vimentin were verified by Western blot (Figure 4B), which were similar to qRT-PCR results.

### PODNL1 promoted cell proliferation and migration via Akt/mTOR pathway

**Figure 5:**
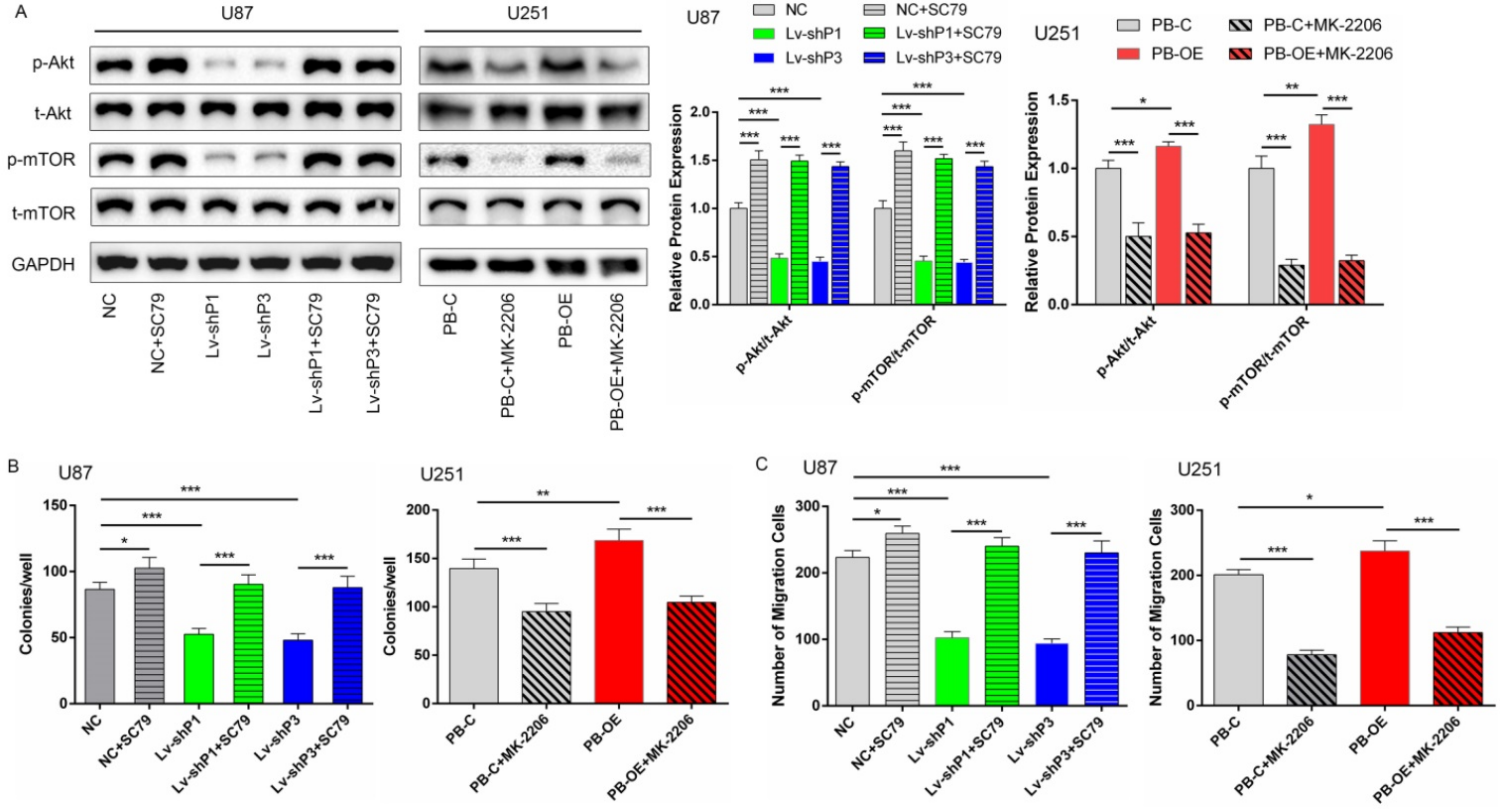
** PODNL1 aggravated glioma progression via Akt/mTOR pathway. A,** The protein levels of phospho-Akt (p-Akt), total-Akt (t-Akt), phosphor-mTOR (p-mTOR), and total-mTOR (t-mTOR) were detected by Western blot in U87 and U251 cells treated with SC79, MK-2206 or DMSO.** B, C,** Effects of proliferation **(B)** and migration **(C)** influenced by PODNL1 could be partially rescued. (*P < 0.05, **P < 0.01, ***P < 0.001).

The above results showed that PODNL1 could promote cell proliferation, migration and inhibit EMT in glioma cells. Currently, accumulating studies have reported that Akt/mTOR pathway directly regulates malignant behaviors in glioma 12, 13. Thus, we supposed that PODNL1 aggravated malignant behaviors through Akt/mTOR pathway in glioma cells. Western blot revealed that silencing of PODNL1 decreased phosphorylation of Akt and mTOR (Figure S4A). Similarly, PODNL1 overexpression showed the opposite trend (Figure S4A). Next, rescue experiments were conducted to validate these findings. Re-activating Akt/mTOR pathway using 8 µg/ml SC79 (Figure 5A), a novel brain-penetrable Akt activator, partially rescued the proliferation and migration inhibition effects of PODNL1 knockdown (Figure 5B, C), evaluated by colony formation and Transwell assays. Conversely, MK-2206, a specific Akt inhibitor, effectively inactivated Akt/mTOR pathway (Figure 5A), which suppressed cell proliferation and migration promoted by PODNL1 overexpression (Figure 5B, C). Collectively, our results demonstrated that PODNL1 stimulated cell proliferation and migration via regulating Akt/mTOR axis.

## Discussion

The SLRP family, which participate in matrix structural organization as ubiquitous ECM components, comprise five classes (I-V) according to the numbers of exons, interspaced amino acid residues and LRR motifs 14-16. Accumulating evidence suggests that the SLRP family are associated with a wide spectrum of biological processes 17, such as embryogenesis 18, regeneration 19 and tumor progression 20. The class V SLRPs bind collagen type I and suppress cell growth and migration 21. PODNL1, as a member of class V, is firstly reported in osteoblastic cells and newly formed bones 22. Currently, several studies have indicated that PODNL1 acts as a biomarker of prognosis in ovarian cancer 23. Importantly, Yan *et al* 3 have reported PODNL1 is overexpressed in high-grade glioma and Shergalis *et al* 4 have indicated that PODNL1 expression is negatively correlated with overall survival. In addition, several members of the SLRP family also function as tumor suppressors via regulating PI3K/Akt/mTOR pathway 24-26. Nevertheless, both the biological function and mechanism of PODNL1 in glioma remain unclear.

In our study, we found that PODNL1 expression was negatively correlated with the prognosis of glioma patients from both TCGA and CGGA databases. In addition, PODNL1 knockdown suppressed glioma cell proliferation, migration, as well as inducing apoptosis. Moreover, most of these phenotypes showed the opposite trend after PODNL1 overexpressed in glioma. Furthermore, we also demonstrated that PODNL1 was essential for the expression of the mesenchymal-related biomarkers in glioma cells, evidenced by remarkably promoting of vimentin, N-cadherin, snail and fibronectin expression after PODNL1 overexpressed, and vice versa.

It is well accepted that PI3K/Akt/mTOR pathway involves cell survival, proliferation and cytoskeletal organization 27-29. mTOR is a 289 kDa serine/threonine protein kinase localized in two structurally and functionally distinct multiprotein complexes known as mTORC1 and mTORC2 30. Activation of mTORC1 downstream of PI3K and Akt drives glioma cell growth by controlling numerous processes that regulate protein synthesis and degradation 31. On the other hand, mTORC2 also increases cellular proliferation and survival through the regulation of protein kinases, including Akt 32, placing mTOR on both sides of the Akt signaling hub. Previous studies have revealed that 6/18 (decorin, biglycan, asporin, fibromodulin, PRELP, osteoglycin) of SLRP members function via regulating PI3K/Akt/mTOR pathway 24, 33-37, which suggests that the potential relationship between SLRP and PI3K/Akt/mTOR axis. In the present, PODNL1 knockdown inactivated Akt/mTOR signaling pathway and overexpression showed the opposite results. Moreover, the glioma cell growth and migration suppressed by PODNL1 knockdown could be partially rescued using Akt activator. Similarly, cell proliferation and migration induced by PODNL1 overexpression could be partially reversed after Akt/mTOR pathway was inhibited by MK-2206.

There are several potential limitations to this study. First, glioma tissues and patients' outcomes in our single center should be collected to further confirm the association between PODNL1 and overall survival. Second, we failed to identify a specific complex or protein which PODNL1 directly interacted with. The following study will focus on these limitations.

## Conclusion

In summary, PODNL1 expression was negatively correlated with the prognosis of glioma patients, as well as along with the WHO grade II-IV. To our best knowledge, this was the first investigation which demonstrated that the biological function of PODNL1 in glioma, and indicated that PODNL1 promoted glioma progression partially via Akt/mTOR pathway. Our study provided a novel biomarker and a potential therapeutic target for glioma.

## Supplementary Material

Supplementary figures.

Supplementary tables.

## Figures and Tables

**Table 1:** The specific primer sequences

Primer	Sequence
PODNL1	F: 5'-CGTGGTCTGCGCTATTTGTTG-3';
	R: 5'-GCCAGGTTAAGCTCCGTCAG-3'.
E-cadherin	F: 5'-GTAGGAAGGCACAGCCTGTC-3';
	R: 5'-CAGCAAGAGCAGCAGAATCA-3'.
N-cadherin	F: 5'-GAGCATGCCAAGTTCCTGAT-3';
	R: 5'-TGGCCACTGTGCTTACTGAA-3'.
Vimentin	F: 5'-CTGCAGGACTCGGTGGACTT-3';
	R: 5'-GAAGCGGTCATTCAGCTCCT-3'.
Snail	F: 5'-TCTGAGGCCAAGGATCTCCA-3';
	R: 5'-GTGGCTTCGGATGTGCATCT-3'.
Fibronectin	F: 5'-ACCTGGAGGAGACCACATGA-3';
	R: 5'-CCATCATCCAGCCTTGGTAG-3'.
GADPH	F: 5'-GATCATCAGCAATGCCTCCT-3';
	R: 5'-TGAGTCCTTCCACGATACCA-3'.

## Abbreviations

PODNL1: podocan-like protein 1
SLRP: small leucine-rich proteoglycan
GEPIA: gene expression profiling interactive analysis
CGGA: Chinese glioma genome atlas
TCGA: The cancer genome atlas
IDH: isocitrate dehydrogenase
DMSO: dimethyl sulfoxide
DMEM: Dulbecco's modified Eagle's medium
FBS: fetal bovine serum
BSA: bovine serum albumin
SD: standard deviation
H&E: hematoxylin-eosin staining
IHC: immunohistochemistry
DAB: 3,3'-diaminobenzidine
mTOR: mammalian target of rapamycin

## References

[1] Lapointe S, Perry A, Butowski NA (2018). Primary brain tumours in adults. Lancet.

[2] Louvel G, Metellus P, Noel G, Peeters S, Guyotat J, Duntze J (2016). Delaying standard combined chemoradiotherapy after surgical resection does not impact survival in newly diagnosed glioblastoma patients. Radiother Oncol.

[3] Yan Y, Zhang L, Xu T, Zhou J, Qin R, Chen C (2013). SAMSN1 is highly expressed and associated with a poor survival in glioblastoma multiforme. PLoS One.

[4] Shergalis A, Bankhead A 3rd, Luesakul U, Muangsin N, Neamati N (2018). Current Challenges and Opportunities in Treating Glioblastoma. Pharmacol Rev.

[5] Tang Z, Li C, Kang B, Gao G, Li C, Zhang Z (2017). GEPIA: a web server for cancer and normal gene expression profiling and interactive analyses. Nucleic Acids Res.

[6] Xu C, Liu X, Geng Y, Bai Q, Pan C, Sun Y (2017). Patient-derived DIPG cells preserve stem-like characteristics and generate orthotopic tumors. Oncotarget.

[7] Kokovay E, Wang Y, Kusek G, Wurster R, Lederman P, Lowry N (2012). VCAM1 is essential to maintain the structure of the SVZ niche and acts as an environmental sensor to regulate SVZ lineage progression. Cell Stem Cell.

[8] Hu XL, Chen G, Zhang S, Zheng J, Wu J, Bai QR (2017). Persistent Expression of VCAM1 in Radial Glial Cells Is Required for the Embryonic Origin of Postnatal Neural Stem Cells. Neuron.

[9] Fischer AH, Jacobson KA, Rose J, Zeller R (2008). Hematoxylin and eosin staining of tissue and cell sections. CSH Protoc.

[10] Metellus P, Coulibaly B, Colin C, de Paula AM, Vasiljevic A, Taieb D (2010). Absence of IDH mutation identifies a novel radiologic and molecular subtype of WHO grade II gliomas with dismal prognosis. Acta Neuropathol.

[11] Sabha N, Knobbe CB, Maganti M, Al Omar S, Bernstein M, Cairns R (2014). Analysis of IDH mutation, 1p/19q deletion, and PTEN loss delineates prognosis in clinical low-grade diffuse gliomas. Neuro Oncol.

[12] Li M, Cheng J, Ma Y, Guo H, Shu H, Huang H (2020). The histone demethylase JMJD2A promotes glioma cell growth via targeting Akt-mTOR signaling. Cancer Cell Int.

[13] Shahcheraghi SH, Tchokonte-Nana V, Lotfi M, Lotfi M, Ghorbani A, Sadeghnia HR (2020). Wnt/beta-catenin and PI3K/Akt/mTOR Signaling Pathways in Glioblastoma: Two Main Targets for Drug Design: A Review. Curr Pharm Des.

[14] Listik E, Azevedo Marques Gaschler J, Matias M, Neuppmann Feres MF, Toma L, Raphaelli Nahas-Scocate AC (2019). Proteoglycans and dental biology: the first review. Carbohydr Polym.

[15] Iozzo RV (1997). The family of the small leucine-rich proteoglycans: key regulators of matrix assembly and cellular growth. Crit Rev Biochem Mol Biol.

[16] Iozzo RV (1999). The biology of the small leucine-rich proteoglycans. Functional network of interactive proteins. J Biol Chem.

[17] Couchman JR (2010). Transmembrane signaling proteoglycans. Annu Rev Cell Dev Biol.

[18] Wilda M, Bachner D, Just W, Geerkens C, Kraus P, Vogel W (2000). A comparison of the expression pattern of five genes of the family of small leucine-rich proteoglycans during mouse development. J Bone Miner Res.

[19] Pang X, Dong N, Zheng Z (2019). Small Leucine-Rich Proteoglycans in Skin Wound Healing. Front Pharmacol.

[20] Appunni S, Anand V, Khandelwal M, Gupta N, Rubens M, Sharma A (2019). Small Leucine Rich Proteoglycans (decorin, biglycan and lumican) in cancer. Clin Chim Acta.

[21] Shimizu-Hirota R, Sasamura H, Kuroda M, Kobayashi E, Saruta T (2004). Functional characterization of podocan, a member of a new class in the small leucine-rich repeat protein family. FEBS Lett.

[22] Mochida Y, Kaku M, Yoshida K, Katafuchi M, Atsawasuwan P, Yamauchi M (2011). Podocan-like protein: a novel small leucine-rich repeat matrix protein in bone. Biochem Biophys Res Commun.

[23] Teng C, Zheng H (2017). Low expression of microRNA-1908 predicts a poor prognosis for patients with ovarian cancer. Oncol Lett.

[24] Xu T, Zhang R, Dong M, Zhang Z, Li H, Zhan C (2019). Osteoglycin (OGN) Inhibits Cell Proliferation and Invasiveness in Breast Cancer via PI3K/Akt/mTOR Signaling Pathway. Onco Targets Ther.

[25] Jiang R, Hao P, Yu G, Liu C, Yu C, Huang Y (2019). Kaempferol protects chondrogenic ATDC5 cells against inflammatory injury triggered by lipopolysaccharide through down-regulating miR-146a. Int Immunopharmacol.

[26] Fang D, Lai Z, Wang Y (2019). Overexpression of Biglycan is Associated with Resistance to Rapamycin in Human WERI-Rb-1 Retinoblastoma Cells by Inducing the Activation of the Phosphatidylinositol 3-Kinases (PI3K)/Akt/Nuclear Factor kappa B (NF-kappaB) Signaling Pathway. Med Sci Monit.

[27] Hemmings BA, Restuccia DF (2015). The PI3K-PKB/Akt pathway. Cold Spring Harb Perspect Biol.

[28] Gulati N, Karsy M, Albert L, Murali R, Jhanwar-Uniyal M (2009). Involvement of mTORC1 and mTORC2 in regulation of glioblastoma multiforme growth and motility. Int J Oncol.

[29] Akhavan D, Cloughesy TF, Mischel PS (2010). mTOR signaling in glioblastoma: lessons learned from bench to bedside. Neuro Oncol.

[30] Janku F, Yap TA, Meric-Bernstam F (2018). Targeting the PI3K pathway in cancer: are we making headway?. Nat Rev Clin Oncol.

[31] Wood AR, Esko T, Yang J, Vedantam S, Pers TH, Gustafsson S (2014). Defining the role of common variation in the genomic and biological architecture of adult human height. Nat Genet.

[32] Sarbassov DD, Guertin DA, Ali SM, Sabatini DM (2005). Phosphorylation and regulation of Akt/PKB by the rictor-mTOR complex. Science.

[33] Hu Y, Sun H, Owens RT, Wu J, Chen YQ, Berquin IM (2009). Decorin suppresses prostate tumor growth through inhibition of epidermal growth factor and androgen receptor pathways. Neoplasia.

[34] Iacob S, Cs-Szabo G (2010). Biglycan regulates the expression of EGF receptors through EGF signaling pathways in human articular chondrocytes. Connect Tissue Res.

[35] Wang L, Wu H, Wang L, Zhang H, Lu J, Liang Z (2017). Asporin promotes pancreatic cancer cell invasion and migration by regulating the epithelial-to-mesenchymal transition (EMT) through both autocrine and paracrine mechanisms. Cancer Lett.

[36] Hu H, Li S, Li J, Huang C, Zhou F, Zhao L (2018). Knockdown of Fibromodulin Inhibits Proliferation and Migration of RPE Cell via the VEGFR2-AKT Pathway. J Ophthalmol.

[37] Ning X, Deng Y (2017). Identification of key pathways and genes influencing prognosis in bladder urothelial carcinoma. Onco Targets Ther.

