# Supplementary material for: PODNL1 promotes cell proliferation and migration in glioma via regulating Akt/mTOR pathway
Source: J Cancer. 2020 Aug 27;11(21):6234–42. doi: 10.7150/jca.46901 (PMC7532506; doi:10.7150/jca.46901)
Supplement: Supplementary file 1 — Supplementary figures. [file jcav11p6234s1.pdf]

Supplementary Figure 1

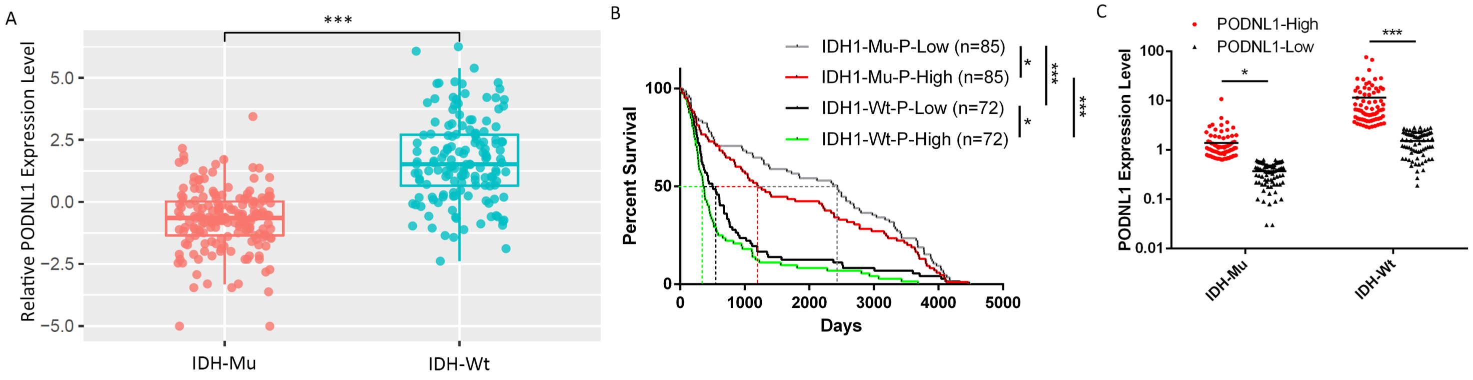

Figure S1. Clinical significance of PODNL1 expression between IDH wildtype (IDH-Wt) and mutant (IDH-Mu) group. A, Comparison of PODNL1 expression between IDH-Wt and IDH-Mu groups. **B,** High PODNL1 expression predicted shorter overall survival in both IDH-Wt and IDH-Mu groups. **C,** The PODNL1 expression showed a significant difference between PODNL1 high and low groups in **Supplementary Figure 1B**. (*P < 0.05, ***P < 0.001)

Supplementary Figure 2

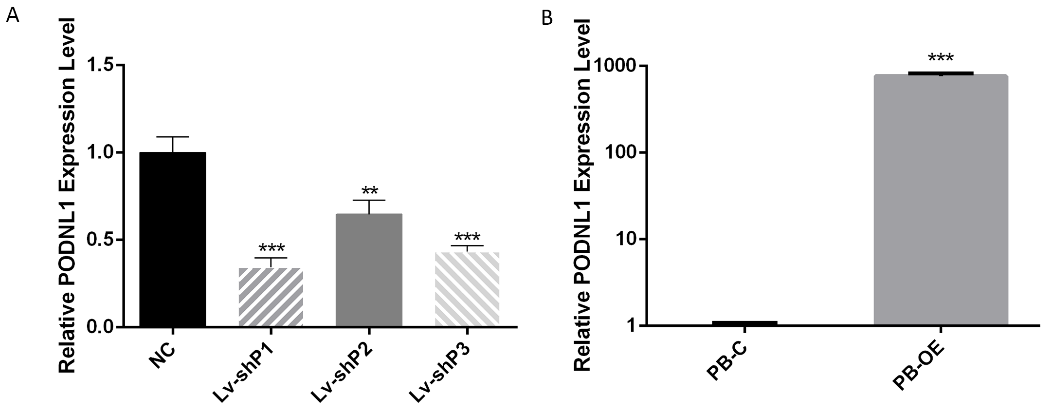

Figure S2. PODNL1 mRNA expression evaluated by qRT-PCR. A, The PODNL1 expression of the U87 cell line transfected with NC and three PODNL1 targeting shRNAs. **B,** The PODNL1 expression of U251 cell line transfected with PB-C and PB-OE. (**$P < 0.01$, ***$P < 0.001$)

Supplementary Figure 3

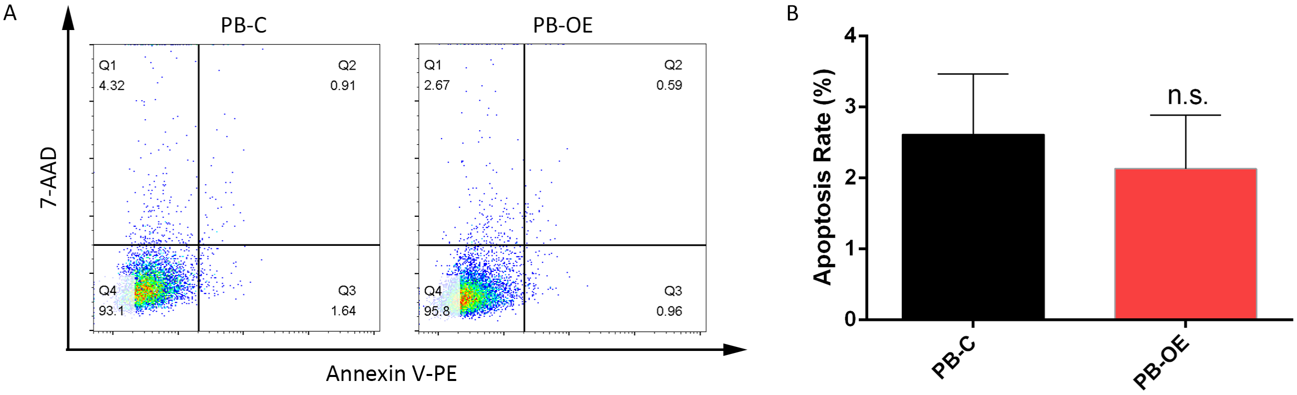

Figure S3. PODNL1 overexpression did not induce the apoptotic change in U251 glioma cells. A, Representative images indicating apoptosis rate between PB-C and PB-OE groups. **B,** Statistical graph suggested no significant difference (n.s.) between PB-C and PB-OE groups.

Supplementary Figure 4

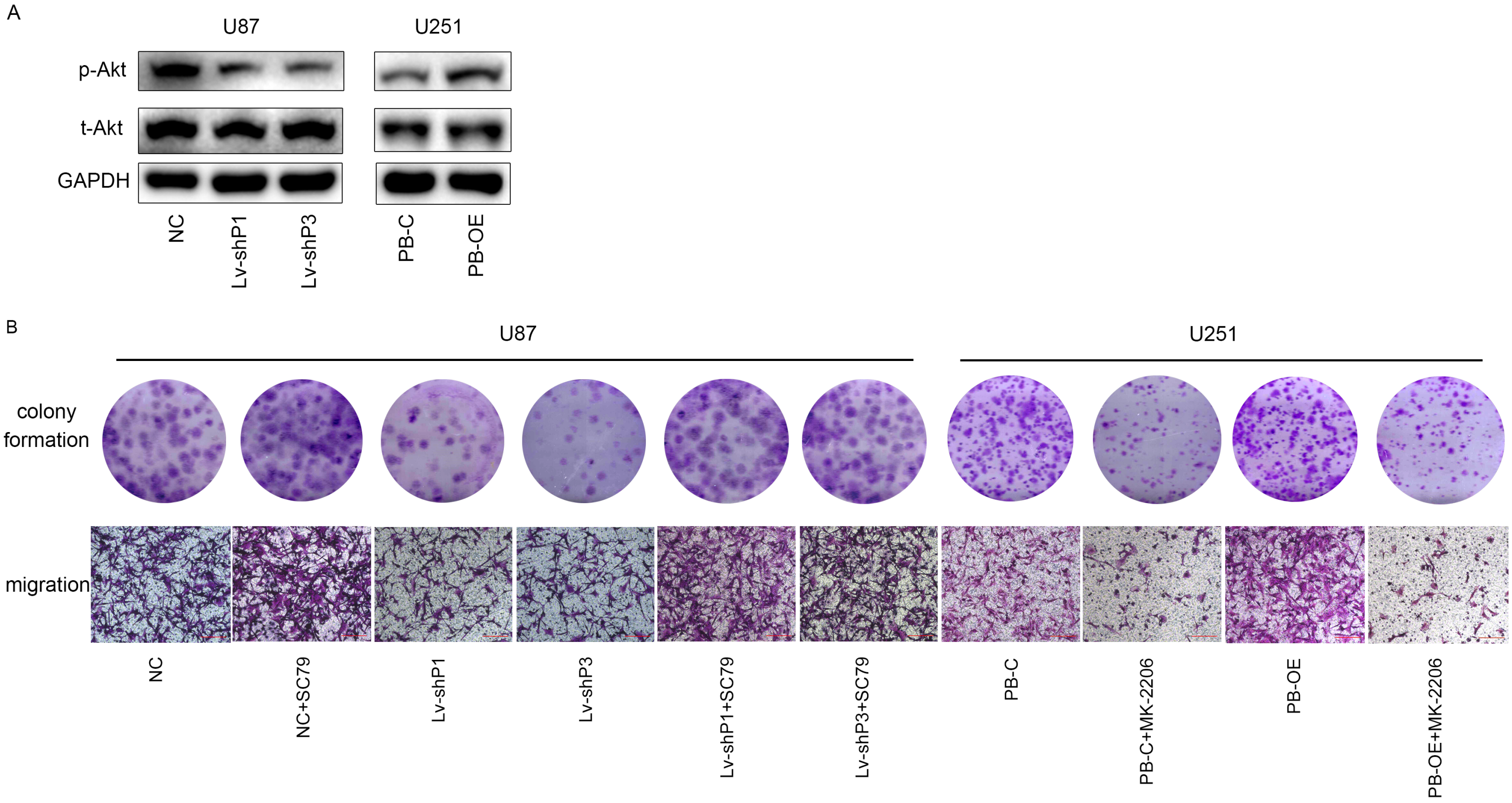

Figure S4. PODNL1 promoted glioma progression via PI3K/Akt pathway. A, The activation status of PI3K/Akt signaling determined by Western blot. **B,** Representative images from colony formation and Transwell assays.
